# The impact of vitamin D status on changes in bone mineral density during treatment with bisphosphonates and after discontinuation following long-term use in post-menopausal osteoporosis

**DOI:** 10.1186/1471-2474-8-3

**Published:** 2007-01-10

**Authors:** Andrew Deane, Leonor Constancio, Ignac Fogelman, Geeta Hampson

**Affiliations:** 1 Department of Chemical Pathology and metabolic bone clinic, St Thomas' Hospital campus, Kings College London, London, UK; 2 Department of Nuclear medicine and metabolic bone clinic, Guy's Hospital campus, Kings College London, London, UK

## Abstract

**Background:**

It is still unclear whether addition of calcium/vitamin D supplements leads to an incremental benefit in patients taking bisphosphonates and whether achievement of serum level of 25 (OH) vitamin D of at least 70 nmol/L has an impact on the skeletal response to bisphosphonates. Moreover the maintenance of BMD after bisphosphonates withdrawal with the continuation of calcium/vitamin D supplements only, remains uncertain. The aims were to assess the impact of vitamin D status on changes in bone mineral density (BMD) in firstly patients with post-menopausal osteoporosis on bisphosphonates and secondly following discontinuation of bisphosphonates after long-term use.

**Methods:**

Two patient groups were recruited. The first study population comprised of 112 women treated with a bisphosphonate. The second study population consisted of 35 women who had been on bisphosphonates for > 5 years in whom the treatment agent was discontinued. Baseline BMD, changes in BMD following treatment, duration of treatment, serum 25 (OH) vitamin D, parathyroid hormone (PTH), urine C-terminal telopeptides of type 1 collagen (CTX) were obtained on the study participants.

**Results:**

In the first study group, subjects with serum vitamin D concentrations (> 70 nmol/L) had a significantly lower serum PTH level (mean [SEM] 41 [2] ng/L). PTH concentrations of 41 ng/L or less was associated with a significantly higher increase in BMD at the hip following treatment with bisphosphonates compared to patients with PTH > 41 ng/L (2.5% [0.9] v/s -0.2% [0.9], P = 0.04). In the second study group, discontinuation of bisphosphonate for 15 months after long-term treatment did not result in significant bone loss at the lumbar spine and total hip, although a trend towards gradual decline in BMD at the femoral neck was observed.

**Conclusion:**

the data suggest that optimal serum 25 (OH) vitamin D concentration may lead to further reduction in bone loss at the hip in patients on bisphosphonates. A prospective controlled trial is needed to evaluate whether the response to bisphosphonates is influenced by vitamin D status. BMD is preserved at the lumbar spine and total hip following discontinuation of bisphosphonate for a short period following long-term treatment, although a gradual loss occurs at the femoral neck.

## Background

Bisphosphonates are widely used in the treatment of osteoporosis and prevention of osteoporosis-related fractures. The efficacy of these anti-resorptive agents has been extensively studied in several randomised controlled trials [1,2]. In all published clinical trials, calcium and vitamin D supplements have also been used with the bisphosphonates as adjunctive therapy. Large scale studies have shown that vitamin D supplementation between 700 and 800 I.U/day leads to a reduction in the risk of hip fracture and non-vertebral fractures by 30% in adults older than 65 years [3], although recent trials have shown that vitamin D, either alone or in combination with calcium supplementation was ineffective in the primary or secondary prevention of fractures in community-dwelling older people [4,5]. However in the RECORD study [4], concentrations of 25 (OH) vitamin D were measured only in a very small subset of the patients. Therefore the vitamin D status of the majority of participants was unknown. Low serum concentrations of vitamin D is widespread in the U.K [6] and moderate vitamin deficiency in older people results in poor bone and muscle strength [7,8]. Serum 25 (OH) vitamin D concentrations that correlate with clinically significant effects on muscle function and fracture prevention is at least 70 nmol/L [9]. It is still unclear whether addition of calcium/vitamin D supplements leads to an incremental benefit in patients taking bisphosphonates and whether achievement of serum level of vitamin D of at least 70 nmol/L has an impact on the skeletal response to bisphosphonates.

Bisphosphonates including Alendronate, Etidronate and Risedronate have a long retention time in bone and there are some concerns about their long-term effects on skeletal mineralization [10]. Recent studies, including the fracture intervention trial have shown that Alendronate, administered for up to 10 years is safe and has continued benefit on the skeleton [11]. The data also show that discontinuation of long-term (5 years or more) Alendronate therapy results in minimal bone loss at the spine, suggesting persistence of Alendronate's effects on bone. However discontinuation of Alendronate seems to lead to gradual loss of effect at the femoral neck and hip [11] although data outside the confines of the randomised controlled trial setting data are lacking. Thus the partial maintenance of bisphosphonates effects after their withdrawal with the continuation of calcium/vitamin D supplements only remains uncertain in routine clinical practice. This is of practical value particularly in patients where compliance is an issue.

The aim of the study was to (1) determine the effect of optimum serum concentrations (> 70 nmol/L) of 25(OH) vitamin D on the skeletal response in patients treated with bisphosphonates and (2) assess changes in bone mineral density (BMD) following 12–18 months withdrawal of Etidronate or Alendronate in long-term users (> 5 years) with the continuation of calcium/vitamin D supplements in post-menopausal osteoporosis, in the practical setting of a metabolic bone clinic service.

## Methods

### Study Protocol

#### Subjects

The study is in 2 parts and investigates the influence of vitamin D status on BMD response during treatment with bisphosphonates and after withdrawal of bisphosphonates following long-term treatment. The first part of the study consisted of a survey of 112 post-menopausal women seen at the metabolic bone clinic consecutively for review at Guy's Hospital, London U.K. They were all being followed-up for assessment of their treatment response after bisphosphonate treatment had been instituted in the metabolic bone clinic. The subjects were recruited during their follow-up visit from April 2004 to January 2005 at the clinic. BMD had been measured at baseline prior to treatment and at their follow-up visit. The majority of patients had been referred from primary care for the management of post-menopausal osteoporosis. They were all treated with a bisphosphonate with an average duration of treatment of 3.8 years. The percentage change in BMD from baseline following treatment was calculated at the lumbar spine and the total hip following a mean duration of 3.8 years between measurements. Information on the age of menarche, menopausal age, amount of alcohol consumption, smoking habits, level of exercise, family history of osteoporosis, and history of falls and fractures was obtained on all subjects at consultation. None of the subjects were receiving hormone replacement therapy. As part of routine clinical practice, (1) DEXA scans were carried out to measure BMD at the spine and hip (2) X-rays of the thoracolumbar spine were done on all patients to document any vertebral fractures and (3) measurement of serum parathyroid hormone (PTH), 25(OH) vitamin D were carried out on the same day. A random urine sample for the measurement of urine C-terminal telopeptides of type 1 collagen (CTX), a marker of bone resorption, was also obtained on the patients at their clinic visit. The patients demographics are summarised in table 1.

The second part of the study looked at the effect of bisphosphonates withdrawal with the continuation of calcium/vitamin D supplements in subjects who had been on bisphosphonates long-tern (>5 years). The study population in the second part of the study comprised of 35 post-menopausal women aged (mean [SEM]) 66.7 [2.1] years who had been on long-term treatment (6.3 [0.32] years) with a bisphosphonate (Etidronate or Alendronate) and in whom treatment was discontinued for 12–18 months. Of these, 54.3% (n = 19) were on Etidronate (Didronel PMO) and 45.7% (n = 16) on Alendronate (10 mg daily or 70 mg weekly). After withdrawal of bisphosphonate, all patients were maintained on 1 g calcium and 800 I.U cholecalciferol daily (Calcichew D3 Forte, 2 tablets daily). They were seen at 12–18 months (mean 15 months) after bisphosphonate was discontinued when BMD at the spine, hip and femoral neck was re-assessed.

#### Analytical methods

Bone mineral density (BMD) was measured at the lumbar spine and hip by dual-energy X-ray absorptiometry (DEXA) (QDR 4500, Hologic, Waltham, MA, USA). Instrument quality control was assessed by daily scanning of a spine phantom. The co-efficient of variation (C.V) for the *in vivo *BMD measurements were 1 % at the lumbar spine and total hip. Body mass index (BMI) was calculated using weight and height.

Haemoglobin, serum electrolytes, urea, creatinine, albumin, calcium and phosphate were measured by standard laboratory procedures on the Beckman LX 20 analyser (Beckman Coulter, Fulterton, CA, USA). Serum PTH was measured on the Nicholas Advantage (Nicholas Institute, Diagnostic Ltd. Essex, UK) by a chemiluminometric assay. The reference range of the assay is 10–65 ng/L. Serum 25 (OH) vitamin D was determined by radioimmunoassay (RIA) using a commercial kit following a rapid extraction procedure (Diasorin, Still water, Minnesota, USA). The assay reference range is 40–195 nmol/L. The inter-assay CV's for serum PTH and 25 (OH) vitamin D were 4.1% and 8.2% respectively at PTH concentrations of 17 ng/ml and 25 (OH) vitamin D concentrations of 57 nmol/L. Urine CTX was determined on a single random urine sample obtained at the clinic visit by an enzyme-linked immunosorbent assay (Urine Crosslaps, Nordic Bioscience Diagnostics A/S, Denmark). The results were expressed after normalization to creatinine excretion. The reference range derived from pre-menopausal women was 45–1035 μg/mmol creatinine. Inter-assay variation was < 10%.

#### Statistical analysis

Comparisons in absolute and percentage change in BMD between groups based on their vitamin D status and serum PTH concentrations and after discontinuation of long-term bisphosphonates were made using two-tailed t-tests. All values are given as the mean ± SEM, unless stated. Univariate linear regression analyses were carried out between serum PTH and 25 (OH) vitamin D, serum PTH, vitamin D status and changes in lumbar spine and hip BMD. Two-tailed *p*-value of < 0.05 was considered significant.

## Results

### Effect of vitamin D status during bisphosphonate treatment

In the first part of the study, the population comprised of 112 consecutive patients on bisphosphonates. Thirty (27.4%), 64 (57.4%) and 38 (33%) patients had previously been or were still on Etidronate, Alendronate and Risedronate respectively. Serum 25 (OH) vitamin D results were available on 106 subjects only. The serum concentration was less than 70 nmol/L in 53% (n = 56) of patients (mean [SEM] serum concentrations: 50 [1.9] nmol/L. The serum vitamin D concentrations were higher than 70 nmol/L in 47% (n = 50) of subjects (mean [SEM]: 93 [3.3] nmol/L). Ten percent of patients(n = 11) had serum concentrations below 40 nmol/L. PTH concentrations were significantly lower in subjects with optimum serum vitamin D (> 70 nmol/l) (PTH (vitamin D sufficient group): 41 [2] ng/L v/s vitamin D insufficient group: 61.7 [5.3], p < 0.0001). However in a minority of patients (n = 20) with vitamin D concentrations <70 nmol/L (mean [SEM] 54 [2.5] nmol/L), serum PTH was less than 41 ng/L (mean [SEM] 30 [2.8].

**Figure 1:**
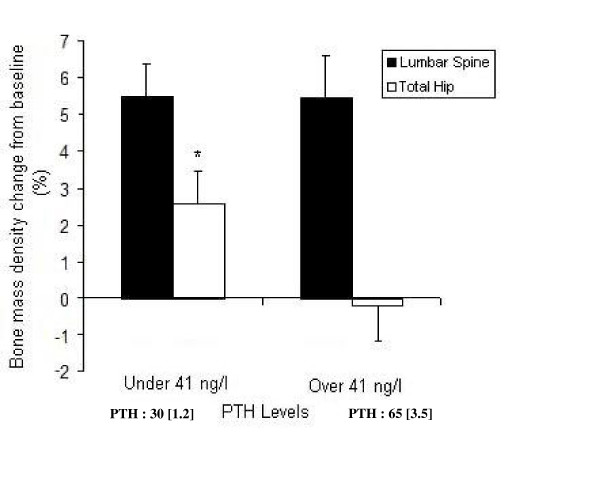
Percentage change in BMD from baseline during bisphosphonate treatment at the lumbar spine and total hip in the study population divided into 2 groups based on their serum PTH concentration (cut-off value: 41 ng/L). A significant difference in % change in BMD at the hip was observed between the 2 groups. Values are shown as the mean [SEM], * p < 0.05.

**Figure 2:**
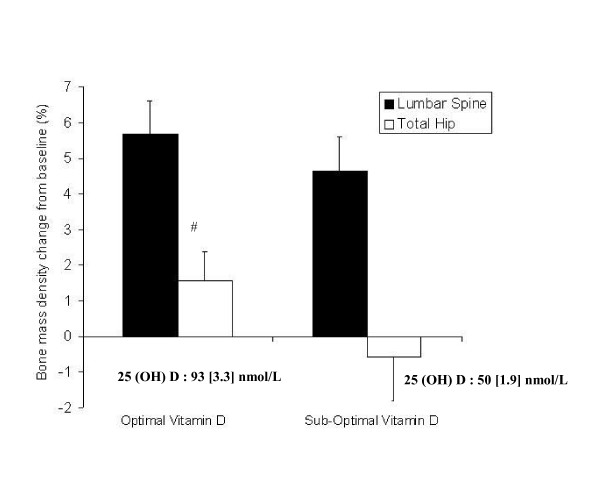
Percentage change in BMD from baseline during bisphosphonate treatment at the lumbar spine and total hip in the study population divided into 2 groups based on their serum 25 (OH) D concentration (cut-off value: 70 nmol/L). The % change in BMD at the hip was less in the group with sub-optimal vitamin D. Values are shown as the mean [SEM], # p = 0.08.

The patients were divided into 2 groups based on their serum PTH concentrations. The cut-off level used was 41 ng/L which corresponded to the mean serum PTH concentrations in the vitamin D sufficient group. PTH was below 41 ng/L in 45% (n = 48) of subjects only (Figure 1). There were no statistically significant differences between the 2 groups with respect to age, BMI, menopausal age, physical activity, type of bisphosphonate and length of treatment. No significant differences in baseline BMD (mean [SD]) at either the lumbar spine or total hip between the 2 sub-groups were observed (patients with serum PTH < 41 ng/L: LS BMD 0.738 [0.098], Total Hip (TH) BMD 0.716 [0.11], serum PTH >41 ng/L: LS BMD .749 [0.12], TH BMD 0.712 [0.01], Patients with serum 25 (OH) vitamin D >70 nmol/L: LS BMD 0.738 [0.1], TH BMD 0.71 [0.12], serum 25 (OH) vitamin D < 70 nmol/L: LS BMD 0.75 [0.12], TH BMD 0.714 [0.09]). There was no significant difference in percentage increase or absolute change in lumbar spine BMD following bisphosphonate treatment between the 2 groups (Patients with serum PTH < 41 ng/L: % change in BMD 5.4 [1.13], serum PTH > 41 ng/L: 5.45 [0.8]). In the patients with lower serum PTH and optimum vitamin D concentrations, hip BMD improved by 2.5%. A significant difference in the changes in BMD at the hip was seen between the 2 groups as shown in Figure 1. The absolute changes in BMD between the 2 groups were also significant (Patients with serum PTH < 41 ng/L: 0.014 [0.006], serum PTH > 41 ng/L: -0.0002 [0.005] g/cm^2^, p < 0.05). A similar trend in the percentage and absolute changes in BMD at the hip was observed when comparing BMD changes between the women according their serum 25 (OH) vitamin D concentrations using the optimum cut-off level of 70 nmol/L (9) (Figure 2), (Patients with serum 25 (OH)D < 70 nmol/L: -0.004 [0.007], serum 25 (OH)D >70 nmol/L: 0.01 [0.004] g/cm^2^, p = 0.06) although the results failed to reach statistical significance. There was no significant difference in urine CTX between the patients based on their PTH concentrations (PTH < 41 ng/L: 148 [29], PTH > 41 ng/L: 162 [23], p = 0.7).

**Figure 3:**
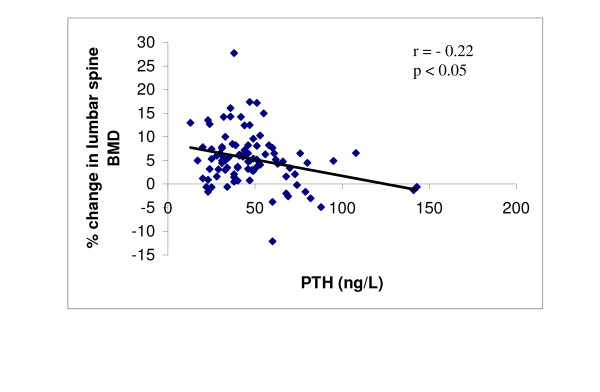
The relationship between percentage change in BMD at the Lumbar spine and PTH concentrations.

**Figure 4:**
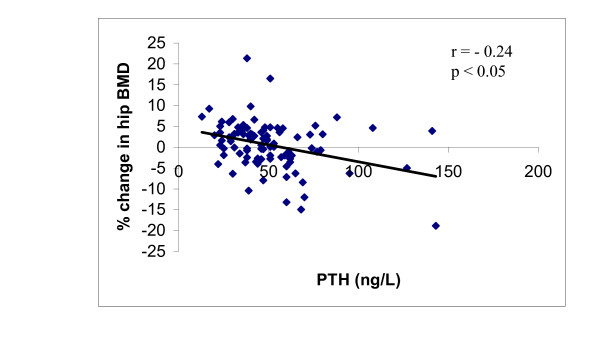
The relationship between percentage change in BMD at the total hip and PTH concentrations.

A strong negative correlation was seen between serum PTH and 25 (OH) vitamin D (r = -0.33, p < 0.0001). Small but significant correlations were observed between the % changes in lumbar spine and hip BMD and PTH concentrations (LS BMD: r = -0.22, p < 0.05, Total hip BMD; r = -0.24, p < 0.05) Figures 3 and 4.

### Effect of bisphosphonate withdrawal with continuation of calcium/vitamin D supplements only on BMD

**Figure 5:**
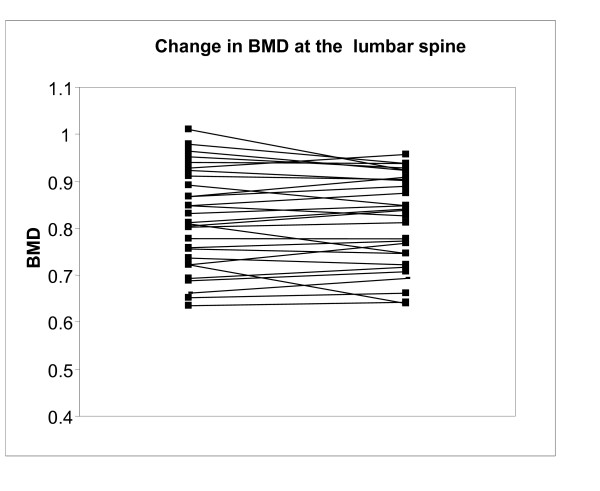
Changes in BMD at the lumbar spine following discontinuation of bisphosphonates for 12–18 months after long-term use.

**Figure 6:**
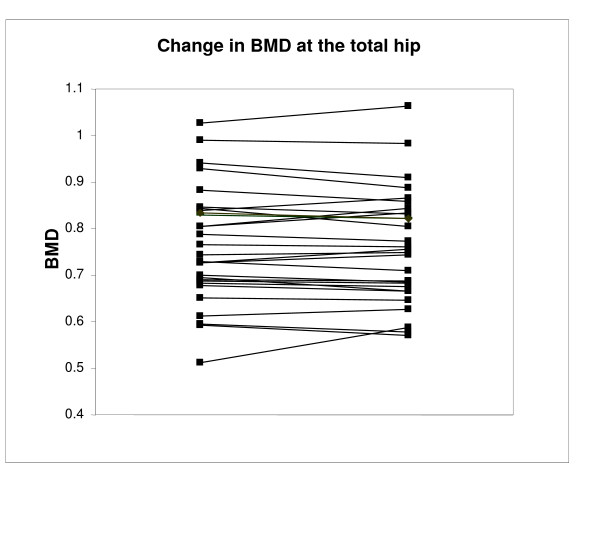
Changes in BMD at the hip following discontinuation of bisphosphonates for 12–18 months after long-term use.

**Figure 7:**
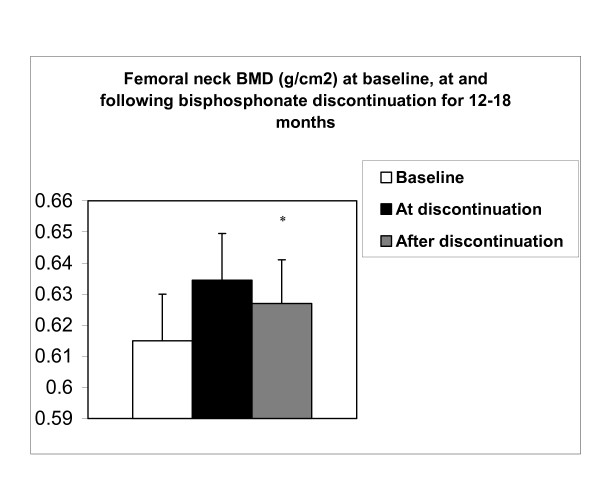
BMD changes at the femoral neck from baseline in the patients at and after discontinuation of long-term bisphosphonates. A downward trend in FN BMD was observed after bisphosphonate withdrawal (* p = 0.094).

In the second study population (n = 35) where bisphosphonates was discontinued for 12–18 months after long-term treatment (> 5 years), BMD increased significantly at all sites from baseline up until the time of discontinuation. The mean percentage increase in BMD during treatment was: LS 8.9 [0.89] %, FN 3.5 [0.06] %, TH 4.4 [0.054] %. Following discontinuation for 12–18 months, there was no significant change in BMD at the lumbar spine and total hip (BMD at discontinuation vs. BMD after discontinuation): LS 0.832 [0.019] vs. 0.831 [0.018] p = 0.809; TH 0.769 [0.021] vs. 0.765 [0.019] p = 0.372 as illustrated in Figures 5 and 6. However, a small reduction in BMD was observed at the femoral neck (FN 0.635 [0.015] vs. 0.627 [0.014] p = 0.094) as shown in Figure 7. Age, body mass index (BMI) and the existence of previous fractures had no effect on the BMD response to bisphosphonate withdrawal. None of the patients sustained a new fracture, although we cannot exclude non-symptomatic vertebral fractures, during the 15 months period off therapy. There was no significant change in BMD after discontinuation in any of the measured sites between patients on etidronate and alendronate, although the patients on Etidronate had been treated for a longer period (p = 0.029) (Table 2).

## Discussion

Serum 25 (OH) vitamin D has been the generally accepted indicator of vitamin D status but there is no uniform consensus on which serum values constitute sufficiency. A number of studies have indicated optimal 25 (OH) vitamin D levels as that required to reduce PTH and bone turnover and values range from 50 nmol/l to 80 nmol/l [12,13]. The threshold for serum 25 (OH) vitamin D concentrations which correlate with optimal bone and musculoskeletal function and reduction in fracture risk in the elderly population has been shown to be at least 70 nmol/l [14].

Recent guidelines recommend that patients receiving treatment for osteoporosis should be calcium and vitamin D replete. Bisphosphonates are well established in the treatment of post-menopausal osteoporosis and fracture prevention. Vitamin D insufficiency may affect the response to anti-resorptive therapy in terms of improvement in BMD as the reduction in bone turnover and mineralization process may be impaired. However, evidence for this is lacking particularly with the newer bisphosphonates and merits investigation in the light of recent epidemiological findings of widespread low serum vitamin D levels in the elderly population [6,7]. There are no clear clinical decision limits for serum 25 (OH) vitamin D concentrations for optimization of bisphosphonates response. Our data show that BMD response to bisphosphonates, at the hip site is impaired in patients who have a PTH level of > 41 ng/L which correlate with a 25 (OH) vitamin D level of < 70 nmol/l. This would be in keeping with loss of predominantly cortical bone which is more commonly associated with high PTH levels. The lack of difference in urine CTX between the 2 groups may be explained, at least in part, by firstly the variability in urinary measurement as we obtained only a single measurement in clinic and secondly bone turnover may be underestimated as the urine samples were non-fasting. Another explanation is that trabecular bone which has a larger surface area than cortical bone is not affected to the same extent. Our findings are in contrast to previous observations with other anti-resorptive agents like raloxifene [15]. Vitamin D insufficiency did not impair the BMD response to raloxifene. This effect may be in part due to raloxifene's ability to increase calcium absorption via its selective oestrogen receptor effects on the intestine, unlike the bisphosphonates. Indeed dietary calcium intake independent of vitamin D status may also regulate PTH. This may explain, at least in part, the findings of a low serum PTH (< 41 ng/ml) in some of our patients with sub-optimum vitamin D (< 70 nmol/L). Bisphosphonates increase bone strength by preventing bone resorption, thus reducing trabecular perforation and cortical porosity and improving bone mineralization as bone remodeling is slowed [16]. Achievement of optimal serum vitamin D concentrations for maximizing bone strength and increases in BMD may be required in patients treated with a bisphosphonate as indicated by our present observations. It may therefore be important for physicians to discuss the additional benefits of vitamin D to such patients. This hypothesis requires further investigations in a prospective controlled trial as there are some limitations to our study, namely the observational study design and the lack of longitudinal 25 (OH) vitamin D and PTH concentrations during the duration of treatment with bisphosphonates.

Bisphosphonates increase BMD by slowing bone turnover, thus allowing secondary mineralisation to progress leading to increased tissue mineral content [17]. However there are some uncertainties about whether the long-term continued mineralisation of older bone increases its strength [18]. For this reason, the long-term use (> 5 years) of bisphosphonates may be detrimental to the skeleton by increasing its brittleness. There is disagreement whether short periods of 1–2 years off therapy is appropriate after every 5 years of treatment in order to prevent bone hypermineralisation and reduction in bone strength. After cessation of bisphosphonates, bone loss occurs less rapidly compared to other anti-resorptive agents such as raloxifene or hormone replacement therapy (HRT) [19], although this remains unclear in the case of Risedronate as the pharmacokinetic properties of this bisphosphonate may be different to Alendronate's. This is because some bisphosphonates in particular Alendronate have a long half-life in bone and may still be biologically active when bone is remodeled, despite discontinuation of treatment [20,21]. Allowing a 'wash-out' period of bisphosphonates from the skeleton may be of clinical importance, particularly as it is now believed, that long-term suppression of bone turnover may be harmful to the skeleton.

The present study suggests that there is good preservation of BMD at the lumbar spine and total hip following a short period of discontinuation of bisphosphonates in long-term users (> 5 years of therapy). Although data were obtained on a relatively small study population, our findings are similar to those of larger clinical trials [11]. BMD at the lumbar spine and total hip did not change after 12–18 months discontinuation of long-term treatment with either Alendronate or Etidronate, provided vitamin D and calcium supplementation is adhered to. There is however less marked preservation of BMD at the femoral neck (FN) although the values remained higher than at baseline as previously described [11]. Our data suggest that accumulation of bisphosphonate in bone after long-term use may result in continued benefits of the drug on BMD, particularly at the spine, after discontinuation for a short period. There is no difference in BMD changes between patients who had been on Etidronate or Alendronate.

## Conclusion

The present study has highlighted 2 important clinical issues in the management of post-menopausal osteoporosis in the practical setting of a metabolic bone clinic. Firstly optimal serum 25 (OH) vitamin D concentrations of 70 nmol/l or higher may be required for maximizing the skeletal response to bisphosphonates. Strategies should be adopted to increase physicians awareness of the rationale for ensuring adequate vitamin D status in women receiving bisphosphonates. Secondly data from the present study suggest that discontinuation of bisphosphonates (Alendronate or Etidronate) for a short period after long-term treatment, whilst continuing with calcium/vitamin D supplements, does not adversely affect BMD at the lumbar spine and total hip. However, the maintenance of BMD after bisphosphonates discontinuation is partial at the femoral neck where a gradual decline in BMD occurred, although the values remained higher than at baseline. Close monitoring may be required, particularly at the femoral neck site and it is important to ensure compliance with calcium/vitamin D supplements.

## Competing interests

The author(s) declare that they have no competing interests.

## Authors' contributions

A.D and L.C contributed to data acquisition/analysis/interpretation. I.F has been involved in revising the manuscript critically for important intellectual content. G.H has contributed to the conception, supervision of A.D sand L.C and drafting the manuscript. All authors have read and approved the final manuscript.

## Pre-publication history

The pre-publication history for this paper can be accessed here:



## Figures and Tables

**Table 1:** Summary of patient demographics and clinical/biochemical characteristice (n = 112). All data are expressed as mean (SEM) and number (percentages).

	**Reference ranges**	
Age (years)	68.4 ± 0.87	
Age at menopause (years)	46.6 ± 0.56	
BMI (kg/m ^2^)	24.2 ± 0.43	
Current smokers (%)	21 (18.6)	
Alcohol intake ≤ 14 units/week (%)	52 (46.4)	
Previous fragility Fracture (s) (%)	68 (60.7)	
Family history of osteoporosis (%)	27 (24.1)	
Family history of fracture (%)	20 (17.9)	
History of falls (%)	31 (27.7)	
Formal exercise (%)	37 (33.0)	
Walking (≥ 30 minutes/day) (%)	26 (50.9)	
Bisphosphonate treatment duration (years)	3.81 ± 0.28	
Serum Calcium (nmol/l)	2.43 ± 0.01	2.2–2.6
Serum 25(OH) vitamin D (nmol/l)	70.6 ± 2.86	40–195
Serum Parathyroid Hormone (ng/l)	51.6 ± 3.12	10–65
Urine CTX (μg/mmol creatinine)	151.3 ± 16.43	45–1035

**Table 2:** Summary of patient characteristics and demographics and change in BMD following discontinuation of etidronate or alendronate.

	**Didronel PMO (n = 19)**	**Fosamax (n = 16)**
Age at Discontinuation (mean [SD])	64.3 [14.41]	69.4 [9.23]
Years on Treatment (mean [SD])	6.8 [2.21]	5.6 [1.03] *
Age at Menarche (mean [SD])	13.0 [1.664]	12.8 [2.49]
Number of years since menopause (mean [SD])	24.7 [9.18]	27.36 [9.04]
Milk Intake ≥ 1/2 pint/day (%)	15.8	6.25
Walking < 30 minutes/day	36.48	25
(%) > 1 hour/day	5.26	12.5
Previous fragility fracture(s) (%)	26.32	37.5
BMI (Kg/m^2^) (mean [SD])	25.47 [6.27]	26.48 [4.67]
Current and Ex-Smokers (%)	36.84	18.75
Alcohol Intake ≤ 14 units/week (%)	52.63	37.5
Positive Family History of Osteoporosis (%)	42.12	25
Lumbar Spine BMD at discontinuation (g/cm^2^) (mean [SD])	0.856 [0.118]	0.761 [0.106]
Lumbar Spine BMD after discontinuation (g/cm^2^) (mean [SD])	0.855 [0.107]	0.769 [0.106]
Total Hip BMD at discontinuation (g/cm^2^) (mean [SD])	0.794 [0.133]	0.739 [0.105]
Total Hip BMD after discontinuation (g/cm^2^) (mean [SD])	0.792 [0.129]	0.736 [0.099]

* p = 0.029

## References

[B1] Black DM, Thompson DE, Bauer DC, Ensrud K, Musliner T, Hochberg MC, Nevitt MC, Suryawanshi S, Cummings SR, Fracture Intervention Trial (2000). Fracture risk reduction with alendronate in women with osteoporosis: The Fracture Intervention Trial. FIT Research Group. J Clin Endocrinol Metab.

[B2] McClung MR, Geusens P, Miller PD, Zippel H, Bensen WG, Roux C, Adami S, Fogelman I, Diamond T, Eastell R, Meunier PJ, Reginster JY, Hip Intervention Program Study Group (2001). Effect of risedronate on the risk of hip fracture in elderly women. Hip Intervention Program Study Group. N Engl J Med.

[B3] Bischoff-Ferrari HA, Willett WC, Wong JB, Giovanni E, Dietrich T, Dawson-Hughes B (2005). Fracture prevention with vitamin D supplementation: a meta-analysis of randomised controlled trials. JAMA.

[B4] The RECORD trial Group (2005). Oral vitamin D3 and calcium for secondary prevention of low-trauma fractures in elderly people (Randomised Evaluation of Calcium Or vitamin D, RECORD): a randomised placebo-controlled trial. Lancet.

[B5] Porthouse J, Cockayne S, King C, Saxon L, Steele E, Aspray T, Baverstock M, Birks Y, Dumville J, Francis R, Iglesias C, Puffer S, Sutcliffe A, Watt I, Torgensen DJ (2005). Randomised controlled trial of calcium and supplementation with cholecalciferol (vitamin D3) for prevention of fractures in primary care. British Medical Journal.

[B6] Hirani V, Primatesta P (2005). Vitamin D concentrations among people aged 65 years and over living in private households and institutions in England: population survey. Age and Ageing.

[B7] Janssen HC, Samson MM, Verhaar HJ (2002). Vitamin D deficiency, muscle function and falls in elderly people. Am J Clin Nutr.

[B8] Reginster JY (2005). The high prevalence of inadequate serum vitamin D levels and implications for bone health. Current Med Res Opinion.

[B9] Vieth R (2005). The role of vitamin D in the prevention of osteoporosis. Ann Med.

[B10] Strewler GJ (2004). Decimal point-Osteoporosis therapy at the 10-year mark. N Engl J Med.

[B11] Bone HG, Hosking D, Devogelaer JP, Tucci JR, Emkey RD, Tonino RP, Rodriguez-Portales JA, Downs RW, Gupta J, Santora AC, Liberman UA, Alendronate Phase III Osteoporosis Treatment Study Group (2004). Ten years' experience with alendronate for osteoporosis in post-menopausal women. N Engl J Med.

[B12] Gallagher SJ, Mc Quillian C, Harkness M, Finlay F, Gallagher AD, Dixon T (2005). Prevalence of vitamin D inadequacy in Scottish adults with non-vertebral fragility fractures. Curr Med Res Opinion.

[B13] Dawson-Hughes B, Heaney RP, Holick HF, Lips P, Meunier PJ, Vieth R (2005). Estimates of optimal vitamin D status. Osteoporos Int.

[B14] Vieth R (2004). Why the optimal requirement for vitamin D_3 _is probably much higher than what is officially recommended for adults. J Steroid Biochem Mol Biol.

[B15] Antoniucci DM, Vittinghoff E, Blackwell T, Black DM, Sellmeyer DE (2005). Vitamin D insufficiency does not affect bone mineral density response to Raloxifene. J Clin Endocrinol Metab.

[B16] Boivin GY, Chavassieux PM, Santora AC, Yates J, Meunier PJ (2000). Alendronate increases bone strength by increasing the mean degree of mineralization of bone tissue in osteoporotic women. Bone.

[B17] Boivin G, Meunier PJ (2002). Changes in bone remodeling rate influence the degree of minaralisation of bone. Connect Tissue Res.

[B18] Odvina CV, Zerwech JE, Rao SD, Maalouf N, Gottschalk FA, Pak CYC (2005). Severely suppressed bone turnover: a potential complication of alendronate therapy. J Clin Endo Metab.

[B19] Seeman E, Eisman JA (2004). Treatment of osteoporosis: why, whom, when and how to treat. MJA.

[B20] Ensrud KE, Barrett-Connor EL, Schwart ZA, Santora AC, Bauer DC, Suryawanshi S, Feldstein A, Haskell WL, Hochberg MC, Torner JC, Lombardi A, Black DM, Fracture Intervention Trial Long-Term Extension Research Group (2004). Randomised trial of effect of alendronate continuation versus discontinuation in women with low BMD: results from the fracture intervention trial long-term extension. J Bone Miner Res.

[B21] Miller PD, Watts NB, Licata AA, Harris ST, Genant HK, Wasnich RD, Ross PD, Jackson RD, Hoseyni MS, Schoenfeld SL, Valent DJ, Chesnut CH (1997). Cyclical etidronate in the treatment of post-menopausal osteoporosis: efficacy and safety after seven years of treatment. Am J Med.

